# Oral Exposure to Genistin, the Glycosylated Form of Genistein, during Neonatal Life Adversely Affects the Female Reproductive System

**DOI:** 10.1289/ehp.0900923

**Published:** 2009-07-27

**Authors:** Wendy N. Jefferson, Daniel Doerge, Elizabeth Padilla-Banks, Kellie A. Woodling, Grace E. Kissling, Retha Newbold

**Affiliations:** 1 Laboratory of Reproductive and Developmental Toxicology and; 2 Laboratory of Molecular Toxicology, National Institute of Environmental Health Sciences, National Institutes of Health, Department of Health and Human Services, Research Triangle Park, North Carolina, USA; 3 National Center for Toxicological Research, U.S. Food and Drug Administration, Jefferson, Arkansas, USA; 4 Biostatistics Branch and; 5 National Toxicology Program, National Institute of Environmental Health Sciences, National Institutes of Health, Department of Health and Human Services, Research Triangle Park, North Carolina, USA

**Keywords:** development, diethylstilbestrol, endocrine disruptors, environmental estrogen, isoflavone, ovary

## Abstract

**Background:**

Developmental exposure to environmental estrogens is associated with adverse consequences later in life. Exposure to genistin (GIN), the glycosylated form of the phytoestrogen genistein (GEN) found in soy products, is of concern because approximately 20% of U.S. infants are fed soy formula. High circulating levels of GEN have been measured in the serum of these infants, indicating that GIN is readily absorbed, hydrolyzed, and circulated.

**Objectives:**

We investigated whether orally administered GIN is estrogenic in neonatal mice and whether it causes adverse effects on the developing female reproductive tract.

**Methods:**

Female CD-1 mice were treated on postnatal days 1–5 with oral GIN (6.25, 12.5, 25, or 37.5 mg/kg/day; GEN-equivalent doses), oral GEN (25, 37.5, or 75 mg/kg/day), or subcutaneous GEN (12.5, 20, or 25 mg/kg/day). Estrogenic activity was measured on day 5 by determining uterine wet weight gain and induction of the estrogen-responsive gene lactoferrin. Vaginal opening, estrous cyclicity, fertility, and morphologic alterations in the ovary/reproductive tract were examined.

**Results:**

Oral GIN elicited an estrogenic response in the neonatal uterus, whereas the response to oral GEN was much weaker. Oral GIN altered ovarian differentiation (i.e., multioocyte follicles), delayed vaginal opening, caused abnormal estrous cycles, decreased fertility, and delayed parturition.

**Conclusions:**

Our results support the idea that the dose of the physiologically active compound reaching the target tissue, rather than the administered dose or route, is most important in modeling chemical exposures. This is particularly true with young animals in which phase II metabolism capacity is underdeveloped relative to adults.

Exposure to environmental estrogens during critical developmental windows has well- documented adverse consequences on males and females of many species, including rodents and humans [National Institutes of Health (NIH) 1999; Palmlund 1996]. One group of environmental endocrine disruptors that is currently receiving significant attention is the naturally occurring phytoestrogens (for review, see Rozman et al. 2006a, 2006b). These compounds are readily available in the diet, particularly in soy products (Adlercreutz et al. 1999; Lapcik et al. 1998; Whitten et al. 1995). The major class of phytoestrogens found in soy is isoflavones; the phytoestrogen in that class that has received the most attention is genistein (GEN). GEN is primarily present in its glycosylated forms, with genistin (GIN), the 4′-β-d-glucoside, being predominantly found in most soy products, including soy-based infant formulas, where GIN makes up > 65% of the isoflavone content (Setchell et al. 1997). Infants consuming soy-based formulas have high circulating levels of GEN, the primary metabolite and aglycone form of GIN, ranging from 1.4 to 4.5 μM (381–1,224 ng/mL) (Setchell et al. 1997); a recent study reported the 25th, 50th, and 75th quartiles in serum as 405.3, 890.7, and 1455.1 ng/mL (1.5, 3.3, and 5.4 μM, respectively) (Cao et al. 2009), indicating that GIN is readily absorbed and hydrolyzed to the aglycone form, GEN.

Studies using experimental rodent models have shown that neonatal exposure to GEN by subcutaneous (sc) injections caused adverse consequences on the female rodent reproductive system, including altered ovarian differentiation, altered estrous cyclicity, subfertility/infertility, and reproductive tract cancer (Chen et al. 2007; Delclos et al. 2009; Jefferson et al. 2002, 2005, 2006; Kouki et al. 2003; Lewis et al. 2003; Nagao et al. 2001; National Toxicology Program 2008; Newbold et al. 2001; Nikaido et al. 2004). After treatment with 50 mg/kg/day, the maximum serum circulating level (*C*_max_) of GEN was 6.8 μM (1,836 ng/mL) (Doerge et al. 2002). Although this level of GEN in mouse serum was only slightly higher than reported in infants on soy-based formulas (Cao et al. 2009; Setchell et al. 1997), we documented adverse effects on the reproductive system at this dose and at lower doses of 0.5 and 5 mg/kg GEN (Jefferson et al. 2002, 2005), suggesting that the levels found in human infants consuming soy-based infant formulas have the potential to cause adverse effects. It was not clear, however, whether the route (sc) and form of compound (GEN) administered were appropriate for predicting human health risks.

Because of limited metabolic capacity of the neonate, it was hypothesized that GIN would not be efficiently hydrolyzed to GEN (Rozman et al. 2006a) and, further, that phase II metabolism such as glucuronidation would also be limited (Coughtrie et al. 1988; Doerge et al. 2001; Onishi et al. 1979). Few studies have reported exposing neonatal rodents orally to GIN because of their small size; however, in a pilot study we demonstrated that oral GIN elicited estrogenic activity (Jefferson et al. 2007). Neonatal mice treated orally on days 2–5 with 25 mg/kg GIN showed increased uterine wet weight gain similar to mice that received 20 mg/kg GEN sc, thus indicating that approximately 80% of orally administered GIN reached sufficient circulating levels of the active compound to elicit a biological effect compared with sc GEN (Jefferson et al. 2007). This is important because Rozman et al. (2006a) argued that orally administered GIN, the form available to infants consuming soy formula, was not biologically active as an estrogen and therefore could not cause adverse effects associated with other environmental estrogens.

The purpose of the present study was to determine neonatal estrogenic activity after oral exposure to GIN, as determined by increased uterine wet weight and induction of the estrogen-regulated gene lactoferrin (*LF*), and then compare this response with that of sc GEN exposure. Further, we determined long-term consequences on the developing reproductive system after orally administered GIN, including multioocyte follicles (MOFs), timing of vaginal opening, estrous cyclicity, and reproductive function. The findings of this study show that oral administration of GIN, the route and chemical found in soy products, including soy-based infant formulas, adversely affects the developing reproductive system in mice.

## Materials and Methods

### Animals

Adult female CD-1 [Crl:CD-1 (ICR) BR] mice were obtained from Charles River Breeding Laboratories (Raleigh, NC) and bred to male mice of the same strain at the National Institute of Environmental Health Sciences (NIEHS). Vaginal plug detection was considered day 0 of pregnancy. Pregnant mice were individually housed in ventilated polysulfone cages (Technoplast, Inc., Exton, PA) with hardwood chip bedding under controlled lighting (12/12-hr light/dark cycle) and temperature (21–22°C) conditions. Mice were fed NIH 31 mouse chow (Zeigler Brothers, Gardners, PA)—which was assayed for phytoestrogen content as previously described (Thigpen et al. 1999)—and provided fresh water *ad libitum*. All animals were treated humanely and with regard for alleviation of suffering, and all animal procedures complied with NIEHS/NIH animal care guidelines.

**Figure 1:**
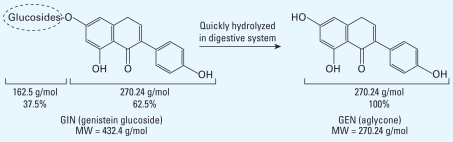
Chemical structures of GIN (oral exposure) and GEN (sc exposure). MW, molecular weight.

Female pups were pooled together, separated by sex, and then randomly standardized to eight female pups per dam; male pups were untreated and used as breeders after reaching adulthood. Female pups were treated by one of two protocols on postnatal days (PNDs) 1–5: *a*) by sc injection with corn oil or GEN (12.5, 20, or 25 mg/kg/day; 98% pure; Sigma Chemical Co., St. Louis, MO) suspended in corn oil (0.02 mL/pup); or *b*) orally with corn oil, GEN (25, 37.5, or 75 mg/kg/day), or GIN (10, 20, 40, or 60 mg/kg/day; 98% pure; Sigma) suspended in corn oil (2.5 μL/g). Oral doses were administered using a pipette inserted inside each pup’s mouth. Pups consumed the dose easily and did not show weight loss, stress, or any other gross toxic effect. Chemical structures of GIN and GEN are shown in Figure 1. Structurally, GIN is composed of GEN and a large sugar group that accounts for 37.5% of its molecular weight. Based on this, we used actual GEN (aglycone) equivalents for dosing with GIN; therefore, 10 mg/kg/day GIN = 6.25 mg/kg/day GEN, 20 mg/kg/day GIN = 12.5 mg/kg/day GEN, 40 mg/kg/day GIN = 25 mg/kg/day GEN, and 60 mg/kg/day GIN 60 = 37.5 mg/kg/day GEN. Throughout the remainder of this article, we refer to these GEN-equivalent doses as GIN 6.25, 12.5, 25, and 37.5 mg/kg.

### Uterotropic bioassay for estrogenicity in neonates

Female pups were treated with oral GIN or GEN or with sc GEN on PNDs 1–5; 4 hr after the last treatment, individual body weights were taken and pups were euthanized by decapitation. Uteri were carefully collected using an Olympus SZX16 dissecting scope (Olympus America, Center Valley, PA), and uterine wet weight was obtained (eight mice per treatment group). Uterine weight was not adjusted for body weight because body weights were not significantly different across treatment groups. Uteri were frozen on dry ice and stored at −80°C.

RNA was isolated from uteri (minimum of four per group) using the RNeasy Mini Kit (Qiagen, Valencia, CA) and then reverse transcribed into cDNA using the First Strand cDNA Synthesis Kit (Invitrogen, Carlsbad, CA). We determined lactoferrin (LF) expression by real-time reverse transcriptase polymerase chain reaction (RT-PCR) as verification of estrogenic activity, as previously reported (Newbold et al. 2007); 18S ribosomal RNA was used for normalization. We determined expression using the mathematical model described by Pfaffl (2001):


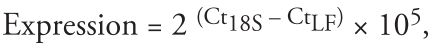


where Ct is the cycle threshold.

### Serum levels of GEN in neonates

Female pups were treated orally with GIN 37.5 mg/kg or GEN 37.5 mg/kg on PNDs 1–5. On PND5 at each time point (0, 0.5, 1, 2, 4, 8, 24, and 48 hr) after the last treatment, four to six individual pups were decapitated and trunk blood was collected. After clotting at room temperature, serum (20–40 μL) was prepared immediately by centrifugation, frozen on dry ice, and stored at −80°C. We determined total GEN content by liquid chromatography with electrospray tandem mass spectrometry (LC-ES/MS/MS) after enzymatic deconjugation and for GEN (aglycone) without deconjugation using aliquots of 10 μL as described previously (Doerge et al. 2002). The limit of detection was approximately 0.03 μM (signal/noise, 3); the inter- and intraday precision and accuracy were 3–8% and 93–96%, respectively (Twaddle et al. 2002). Model-independent pharmacokinetic analysis was performed as previously described (Doerge et al. 2002). Levels of daidzein and equol were also evaluated by LC-ES/MS/MS and found to be consistently undetectable (data not shown).

### Ovarian histology

To determine effects on ovarian development, ovaries were collected prepubertally, after secondary follicle formation but before corpora lutea formation, as previously reported (Jefferson et al. 2002). Female mice treated orally with GIN (0, 6.25, 12.5, 25, or 37.5 mg/kg/day) on PNDs 1–5 were sacrificed at 19 days of age by CO_2_ asphyxiation (eight mice per treatment group). Ovaries were collected and fixed in 10% cold neutral buffered formalin overnight and then changed to cold 70% ethanol. Tissues were then processed for histology, embedded in paraffin, and cut at 5 μm. Three sections from each of three different levels for both ovaries were scored for the presence and numbers of MOFs and any alterations in ovarian histology (total of 18 sections scored per mouse).

### Vaginal opening and estrous cyclicity

Female mice treated orally with GIN (0, 6.25, 12.5, 25, or 37.5 mg/kg) on PNDs 1–5 were weaned at 22 days of age, housed four per cage, and followed daily for vaginal opening (16 mice per treatment group). At 2 months of age, vaginal smears were obtained daily for 2 weeks from half of the mice in each treatment group (eight mice per group). Smears were collected on positively charged slides (Superfrost Plus; Fisher Scientific, Pittsburgh, PA), sprayed with Spraycyte fixative (Fisher), and stained with hematoxylin and eosin (Sigma) to determine the stage of the estrous cycle, as previously described (Champlin et al. 1973). Mice with 3 consecutive days in either diestrus or estrus were considered to have abnormal cycles.

### Fertility assessment

At 2, 4, and 6 months of age, female mice that were used to determine vaginal opening and estrous cyclicity were bred to proven control males of the same strain for a 2-week period (16 mice per treatment group). Females were checked in the morning for vaginal plugs, removed from the male cages, and individually housed until delivery. All females that were not “vaginal plug positive” were removed and then returned to the male cages in the afternoon for a total of six attempts at achieving vaginal plug–positive status. Fertility assessment included the following end points: number of plug-positive females, number of mice with live litters, average number of live pups per litter, and time to delivery.

### Statistical analysis

The data were analyzed using JMP 7 and SAS 9.1 software (both from SAS Institute Inc., Cary, NC). For uterine wet weight and real-time RT-PCR data, we performed analysis of variance, followed by Dunnett’s test. For MOFs, estrous cyclicity, and fertility end points, statistical significance was determined using the nonparametric Mann-Whitney tests or Fisher’s exact test, as appropriate. The Cochran-Armitage trend test was used to test for dose trends in quantal responses such of MOFs. *p*-Values < 0.05 were considered statistically significant.

## Results

### Estrogenic activity in neonates

**Figure 2:**
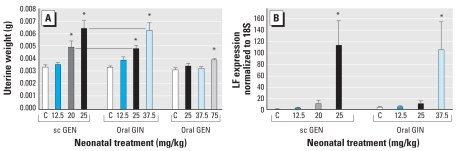
Effects of neonatal treatment with GEN and GIN on female mice on PND5. C, corn oil control. (*A*) Uterotropic bioassay after sc or oral GEN or oral GIN; values shown are mean ± SE uterine wet weight for each treatment group (*n* = 8 mice per group). (*B*) Real-time RT-PCR for LF shown as mean ± SE of LF expression normalized to cyclophilin × 10,000. *p < 0.05, compared with controls, Dunnett’s test.

Female mice treated with sc GEN at 20 and 25 mg/kg/day had increased uterine wet weights at 5 days of age compared with controls (Figure 2A). We also observed increased uterine wet weight in mice treated orally with GIN at 25 and 37.5 mg/kg/day (Figure 2A). Compared with sc GEN, approximately 20–33% more oral GIN was needed to elicit similar uterine wet weight increase compared with controls. We observed no increase after oral GEN 25 or 37.5 mg/kg and only a slight increase at 75 mg/kg/day (Figure 2A), suggesting that much more oral GEN is needed to elicit an estrogenic response compared with either sc GEN or oral GIN.

To investigate functional estrogen activity after sc or oral exposures, we determined mRNA levels of the estrogen-regulated protein LF by real-time RT-PCR. Mice treated by sc GEN 12.5, 20, and 25 mg/kg showed increased *LF* mRNA compared with controls, correlating with increased uterine wet weight at higher doses, resulting in higher expression of LF (Figure 2B). Oral exposure to GIN 12.5, 25, and 37.5 mg/kg showed similar increases in *LF* mRNA compared with controls, which also correlated with increased uterine wet weight (Figure 2B).

### Serum levels of GEN in neonates

**Figure 3:**
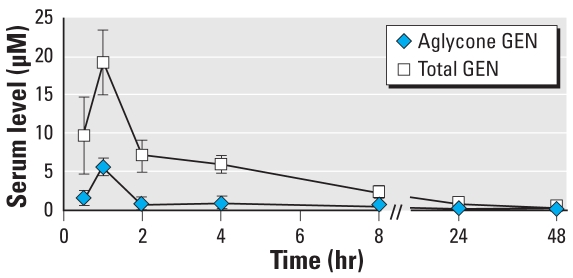
Serum levels of GEN (mean ± SE) after oral exposure to GIN 37.5 mg/kg on PNDs 1–5. Serum was collected at 0.5, 1, 2, 4, 8, 24, and 48 hr after the last treatment (*n* = 4–6 samples per group).

Figure 3 shows the total and aglycone levels of GEN measured in serum after oral treatment with 37.5 mg/kg GIN. These data suggest that the glucoside moiety of GIN is readily cleaved to the aglycone form, GEN, which can either be absorbed into the circulation as the aglycone (*C*_max_ = 5.6 μM) or conjugated by UDP-glucuronosyl transferases in the gut and secreted as conjugated forms into circulation (total *C*_max_ = 19.2 μM). These levels of GEN are higher than those previously reported after sc GEN 50 mg/kg (*C*_max_ = 2.3 μM aglycone and 5.0 μM for total) (Doerge et al. 2002). However, the dose-adjusted area under the curve (AUC) for total GEN is slightly lower after oral GIN (83% of sc GEN) and lower than the AUC for aglycone adjusted for dose (48% of sc GEN; Table 1). These differences are captured by the difference in percent aglycone AUC for sc versus oral administration (22% vs. 13% of total AUC), which reflects the bypass of phase II conjugation in the gut after injection.

The internal exposures after orally administered GEN were much lower compared with oral GIN, measured either as *C*_max_ (~ 1 μM) or as AUC (3.6 μM-hr; Table 1). Therefore, it was not surprising that orally administered GEN did not result in a robust estrogenic response in the neonate; because results of sc administration of GEN have been previously reported (Doerge et al. 2002), in the present study we followed only mice treated by oral GIN for ovarian histology, timing of vaginal opening, estrous cyclicity, and fertility.

### Ovarian histology

To determine the effect of oral GIN on the developing ovary, we evaluated the presence of MOFs in immature mice at 19 days of age. The percentage of MOFs increased with dose of oral GIN (*p* < 0.05, Cochran-Armitage trend test) and were significantly higher than in controls in all dose groups except the lowest dose [see Supplemental Material, Figure 1A, available online (doi:10.1289/ehp.0900923.S1 via http://dx.doi.org/)].

### Vaginal opening and estrous cyclicity

Mice exposed to the highest dose of GIN (37.5 mg/kg) had delayed vaginal opening compared with their age-matched control counterparts, with 50% of the GIN-treated mice achieving vaginal opening 2 days later [see Supplemental Material, Figure 2 (doi:10.1289/ehp.0900923.S1)]. In addition, a few mice in the top two dose groups did not have definitive vaginal opening, even 5 days after the last control mouse exhibited opening. Mice in all other groups appeared to achieve vaginal opening at a similar time as controls.

To determine the effect of oral GIN on estrous cyclicity, vaginal smears were taken for 2 weeks beginning at 2 months of age. None of the mice in the control group had abnormal estrous cycles, whereas 38% of GIN 12.5 mg/kg, 62% of GIN 25 mg/kg, and 88% of GIN 37.5 mg/kg mice had abnormal cycles (*p* < 0.001, Cochran-Armitage trend test), and the highest two groups had cycles significantly different from those of controls [Fisher’s exact test, *p* < 0.05; see Supplemental Material, Figure 1B (doi:10.1289/ehp.0900923.S1)]. Abnormal cycles exhibited by mice were predominantly prolonged time in estrus (with a few mice exhibiting prolonged time in diestrus).

### Fertility assessment

**Figure 4:**
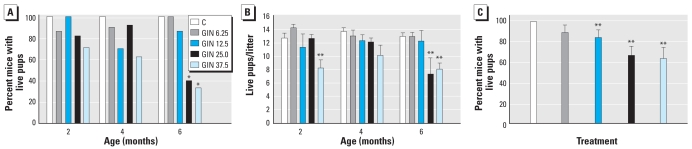
Fertility assessment of mice treated neonatally with oral GIN. C, corn oil control. (*A*) Percentage of vaginal plug–positive mice delivering live pups by age (2, 4, and 6 months). (*B*) Number of live pups per litter (mean ± SE) by age (2, 4, and 6 months). (*C*) Percentage of mice delivering live pups for all ages combined (mean ± SE). *p < 0.05, compared with controls by Fisher’s exact test. **p < 0.05, compared with controls by Mann-Whitney test.

The percentage of oral GIN-treated females that were vaginal plug positive was similar among dose groups at 2, 4, and 6 months of age [Figure 4; see also Supplemental Material, Table 1 (doi:10.1289/ehp.0900923.S1)]. We observed a significant reduction in the number of mice delivering live pups after oral exposure to GIN 25.0 and 37.5 mg/kg at 6 months of age. In addition to fewer pregnant females, mice that did deliver live pups in the GIN 37.5 mg/kg dose had fewer pups at 2 and 6 months of age; the GIN 25 mg/kg group also had fewer pups at 6 months of age.

We also combined all the data across the time points (Figure 4C, Table 2). The percentage of plug-positive females that resulted in litters with live pups was significantly reduced in the oral GIN 12.5, 25.0, and 37.5 mg/kg groups. In addition, the total number of pups produced per dam over the three time points and the average number of pups per litter were significantly reduced in the 37.5 mg/kg group compared with the control group. The average numbers of pups per litter were borderline significantly reduced in the 12.5 and 25.0 mg/kg GIN groups, as was the total number of pups produced in the 12.5 mg/kg group.

**Figure 5:**
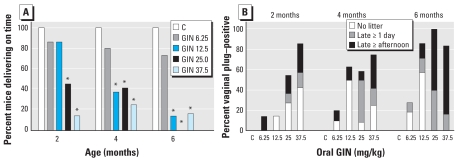
Timing of delivery of live pups. C, corn oil control. (*A*) Percentage of vaginal plug–positive mice delivering on time for each group by age (2, 4, and 6 months). (*B*) Percentage of mice per treatment group by age (2, 4, and 6 months) that delivered in the late afternoon or 1–2 days late, or that delivered no live pups. *p < 0.05 compared with controls by Fisher’s exact test.

The timing of delivery after achieving vaginal plug–positive status is summarized in Figure 5. All control mice (treated with corn oil) delivered their pups early in the morning of gestation day (GD) 19, before 0900 hours. All oral GIN groups had some mice that delivered late in the afternoon of GD19 (between 1400 and 1800 hours) or delivered 1–2 days late, on GD20 or GD21. One mouse in the GIN 6.25 mg/kg group was visibly pregnant but did not deliver by 2 days past expected delivery; after the mouse was euthanized, 16 fully formed pups were found dead. On-time deliveries were significantly reduced after oral GIN 25.0 and 37.5 mg/kg at all time points and oral GIN 12.5 mg/kg at 4 and 6 months of age (*p* < 0.05, Fisher’s exact test). In addition, the percentage of mice with on-time deliveries decreased with increasing dose and increasing time (*p* < 0.05, Cochran-Armitage trend test). Figure 5B shows the percentage of mice with late deliveries (afternoon or 1–2 days late) and no live pups per treatment group over time. More than half of the mice at 4 and 6 months of age in the 12.5, 25, and 37.5 mg/kg groups had either late deliveries or no live pups. We also observed this effect in GIN 6.25 mg/kg mice, with 27% exhibiting late delivery at 6 months of age. After observing this effect at 2 months of age, we took some litter weights at 4 and 6 months of age and calculated the average pup weight per litter. Although there were too few pup weights per treatment group per age to evaluate statistically, the averages combined by category of delivery time suggest increased pup weight with delayed parturition. All litters that were on time (regardless of treatment) had an average pup weight of 1.64 ± 0.03 g (*n* = 28 litters), and litters that were born late in the afternoon were no different, with an average pup weight of 1.65 ± 0.03 g (*n* = 8 litters); however, litters that were born 1 or 2 days late had a significantly increased average pup weight of 1.82 ± 0.04 g (*n* = 7 litters; *p* < 0.05, Student’s *t*-test).

## Discussion

The present study shows that oral exposure to GIN, the glycosylated form of GEN found in soy products, results in estrogenic activity in PND5 female neonates similar to the response to sc GEN. We measured estrogenic activity by increases in uterine wet weight and induction of the estrogen-responsive gene *LF*. This is significant because experimental studies using sc exposure have been criticized for not modeling oral exposure with respect to metabolism and kinetics and were thus dismissed as offering no value for human risk assessment (Rozman et al. 2006a, 2006b). In addition, animal studies using oral dosing of GEN to nursing dams produced very little circulating GEN in neonates, demonstrating that indirect exposure through the dam’s milk is not sufficient to produce levels of GEN that approach levels in human infants consuming soy formula (Doerge et al. 2006; Lewis et al. 2003). Perinatal deficiencies in phase II conjugating activity have also been shown to play a major role in determining internal exposures of rodent fetuses and neonates to the active aglycone form (GEN) (Doerge et al. 2001, 2002). Therefore, questions of relative bioavailability of aglycone versus glucoside forms of isoflavones persist, particularly because soy-based foods and formula contain predominately glucosides (Rozman et al. 2006a, 2006b).

The relative bioavailability of isoflavone glucosides versus aglycones has been studied extensively in rodents and humans, but results have conflicted (Izumi et al. 2000; King et al. 1996; Kwon et al. 2007; Richelle et al. 2002; Rufer et al. 2008; Sepehr et al. 2007; Setchell et al. 2001; Steensma et al. 2006; Zubik and Meydani 2003). It is generally accepted that the aglycone form of isoflavones has the highest estrogenic activity. However, glucosides are quickly hydrolyzed to produce the aglycone form, so administration of either the glucoside or aglycone leads to absorption of the biologically active aglycone. Thus, exposure to GEN is theoretically the sum of the aglycone and respective glucoside concentrations converted on the basis of molecular weight (Rozman et al. 2006a). Absorption of GIN has been demonstrated by showing glucuronidated metabolites of GEN and other GEN metabolites in urine of infants fed soy-based formulas (Hoey et al. 2004). Our study confirms that oral GIN is rapidly hydrolyzed and absorbed into neonatal circulation, which is similar to metabolism in human infants because high circulating levels of GEN are seen after oral ingestion of soy-based infant formulas containing predominantly GIN.

We compared internal exposures to GEN from oral GIN and GEN in the present study with results for sc GEN from our previous study (Doerge et al. 2002). The dose-adjusted AUCs for aglycone and total GEN for oral GIN and sc GEN were remarkably similar (Table 1). The dose-adjusted AUC for GEN aglycone after oral GIN were approximately half those for sc GEN, which reflected the difference in percent aglycone (22% for sc GEN and 13% for oral GIN). Despite this difference, similar magnitudes of effects were observed on uterine weight gain, ovary histology, estrous cyclicity, and fertility. The 50% reduction in aglycone after oral GIN compared with sc GEN, with only a 20% reduction in uterine weight gain, poses interesting questions about the dynamics of circulating levels of GEN and estrogenic activity in target tissues. Whether the peak concentration of GEN or the total exposure to GEN (e.g., AUC) or some combination of the two is the driving factor for estrogenic activity is unknown, but the present data suggest that both might be important. In addition, the glycosylated form can be passively transported across the small intestinal membrane and enter circulation by the sodium-dependent glucose transporter (SGLT1), unlike the mechanism by which the aglycone form is absorbed (i.e., passive diffusion) (Kwon et al. 2007). Detailed information on the metabolism, transport, and absorption of these compounds in neonates is necessary to further understand these results. The lack of measurable levels of daidzein or equol in serum eliminates those compounds as potential sources of estrogenic activity in this model.

Oral exposure to GEN, which has known estrogenic activity in the human adult (Setchell et al. 2001) and in prepubertal and mature rodents (Diel et al. 2004; van Meeuwen et al. 2007), does not have a robust estrogenic activity in the mouse neonate. In the uterotropic bioassay, the highest dose of oral GEN was 75 mg/kg/day, twice the sc GEN or oral GIN dose, yet there was only a slight increase in estrogenic activity in the mouse neonate. Serum circulating levels of GEN after oral exposure to GEN support this lack of biological effect, with very little GEN found in the circulation (only 12% of the sc dose). Lewis et al. (2003) demonstrated similar results in 7 rats. The total AUC for the oral route was approximately 10 times less than the sc treatment, meaning the oral route resulted in only 9% of the circulating levels compared with the sc route.

Although many studies have described a role for phytoestrogens, such as GEN, in influencing hormone-dependent states in adults, there is limited information, especially regarding long-term effects, on infant exposure to soy-based formulas or products. In an epidemiologic study, Cruz et al. (1994) reported increased cholesterol synthesis rates in human infants fed soy-based formulas. Strom et al. (2001) concluded that there was no statistically significant difference in > 30 outcomes measured in young adult women and men (20–34 years of age) who were fed soy-based formula or cow-based formula as infants. However, in women fed soy-based formula, these authors found a significant increase in the number of twin births, duration of the menstrual cycle, and pain associated with the cycle, despite the small cohort size (129 soy-based formula–fed women vs. 269 cow-based formula–fed women). A recent study of human infants fed soy formula, cow milk formula, or breast milk, Bernbaum et al. (2008) found that female infants fed soy formula have re-estrogenization of vaginal cells at 6 months of age; this effect was not observed in the two nonsoy groups. In another recent epidemiology study, Zung et al. (2008) reported that breast tissue is more prevalent in the second year of life in female infants fed soy-based formula than in infants who were breast-fed or fed dairy-based formula. These studies support the idea that soy-based infant formulas exert biological effects, including estrogenic activity.

The present study clearly demonstrates that oral exposure to GIN has adverse consequences on the female reproductive system when exposure occurs during neonatal life. Furthermore, these effects are similar to sc GEN at similar doses (Jefferson et al. 2005). For comparison, the incidence of MOFs after exposure to oral GIN is similar to that after sc GEN, with 37.5% after oral GIN 6.25 mg/kg, 25% after sc GEN 5 mg/kg, and 75% after both oral GIN 37.5 mg/kg and sc GEN 50 mg/kg. Disruptions in estrous cyclicity were also similar after both treatment routes; 88% of the mice in both sc GEN 50 mg/kg and oral GIN 37.5 mg/kg groups exhibited abnormal cycles. We recently demonstrated that the major contribution to infertility in neonatal GEN-treated mice is the oviductal and uterine environment (Jefferson et al. 2009). During the preimplantation period, half of the embryos were lost before the four-cell stage in the oviduct, and the other half were unable to develop properly in the uterus, leading to complete infertility. Although we did not see complete infertility at any dose examined in the present study, observed reduced fertility may result from an insufficient uterine environment. Interestingly, oral GIN 12.5 mg/kg did not exhibit an estrogenic response in the uterus on PND5, yet adverse effects on the reproductive system were seen later in life. Further studies are under way to elucidate mechanisms involved in decreased embryo survival and how developmental exposure to estrogenic chemicals may permanently alter gene expression necessary for maintaining pregnancy.

Delayed parturition was another important finding in the present study. The biological signals for parturition are not fully understood, but several factors, including progesterone levels, placental prostaglandins, and uterine oxytocin receptors, have been identified as playing roles (Cook et al. 2003). Thus, potential reasons for late parturition in neonatal GIN-exposed mice include lack of signaling from the pup (e.g., prostaglandins), elevated progesterone during late pregnancy, or lack of response or signaling from the uterus (e.g., oxytocin receptors) (Cook et al. 2003). Because our previous study showed that high doses of sc GEN render the uterus incapable of supporting pregnancy, it seems reasonable that lower doses might also impair uterine signaling. Studies are under way to understand mechanism(s) responsible for late parturition in these mice.

Although care must be taken in extrapolating data from rodents to humans, important information can be gained from experimental animal studies. In summary, our study demonstrates that exposure to oral GIN during neonatal development results in adverse consequences in the adult female mouse reproductive system. Reduced fertility is seen at GIN doses of 12.5, 25, and 37.5 mg/kg. By comparison, estimated human infant consumption is 6–11 mg/kg isoflavones and 4–7 mg/kg GEN (Setchell et al. 1997, 1998). Although the doses used in our study are slightly higher, similar circulating concentrations of total GEN resulted in mice and humans. To date, the pharmacokinetics of GEN in the serum of infants on soy formulas has not been accurately evaluated; only single-point estimates of the steady-state concentration measured at some unknown time since last feeding have been evaluated [e.g., 2.5 μM (675 ng/mL) mean total GEN in plasma (Setchell et al. 1997), 3.6 μM (972 ng/mL) median total GEN in whole blood (Cao et al. 2009)]. Data on adult human pharmacokinetics suggest that these steady-state estimates are probably much lower than peak levels of GEN. The small margin of difference between circulating GEN levels in infants consuming soy-based infant formula and the levels in neonatal mice that we found to produce significant increases in reproductive toxicity suggest that risks to reproductive health should be carefully considered, especially because an estimated 20–25% of infants in the United States consume soy-based infant formulas and the Committee on Nutrition of the American Academy of Pediatrics found “very limited indications for its use” (Bhatia and Greer 2008). Thus, further studies on reproductive end points in the human population are warranted. Our data also show that perinatal deficiencies in metabolism after oral exposure to GEN can affect internal exposures in ways similar to that of injection, so only the dose of the active chemical that reaches the target tissue is important. The present study further validates the use of sc-injected GEN as a suitable model for oral exposure to GIN in neonatal mice.

## Figures and Tables

**Table 1:** Comparison of serum circulating levels of GEN after sc or oral GEN or oral GIN exposure.

	Total GEN			Aglycone		
Treatment (mg/kg)	AUC	Dose corrected (AUC/dose)	Percent sc	AUC	Dose corrected (AUC/dose)	Percent sc
sc injection						
GEN 50^a^	147	2.9	100	33	0.66	100

Oral exposure						
GIN 37.5	90.4	2.4	83	12.1	0.32	48
GEN 37.5	12.8	0.34	12	3.6	0.10	15

a Data from Doerge et al. (2002) were adjusted for the dose differences in the present study.

**Table 2:** Statistical analysis of fertility end points after oral exposure to GIN (all ages combined).

Outcome, GIN dose (mg/kg)	No.	Mean ± SE	Mann-Whitney *p*-value
No. of litters with live pups per dam			
0	15	1.8 ± 0.2	
6.25	14	1.9 ± 0.2	0.6192
12.5	15	1.4 ± 0.2	0.1114
25.0	15	1.6 ± 0.2	0.3326
37.5	12	1.3 ± 0.2	0.0631

Percent plug positive with live pups			
0	15	100.0 ± 0.0	
6.25	14	89.3 ± 7.7	0.2241
12.5	15	84.4 ± 7.7	0.0498
25.0	15	66.7 ± 8.9	0.0004
37.5	12	63.9 ± 11.0	0.0009

Total no. of live pups per dam			
0	15	23.5 ± 2.6	
6.25	13	26.5 ± 2.6	0.7974
12.5	14	17.9 ± 2.2	0.0570
25.0	13	21.2 ± 2.1	0.3882
37.5	10	13.8 ± 1.9	0.0040

Average no. of live pups per litter			
0	15	13.3 ± 0.5	
6.25	13	13.2 ± 0.4	0.3966
12.5	14	12.0 ± 0.8	0.0540
25.0	13	11.8 ± 0.8	0.0597
37.5	10	9.3 ± 1.0	0.0007

End points for each mouse (all three breeding ages) were combined to examine the overall effect on fertility (number of mice with litters out of 16).
